# Aortic Valve Stenosis and Calcified Abdominal Aortic Stenosis Treated With Lithotripsy-Facilitated TAVR and Aortic Stenting

**DOI:** 10.1016/j.jaccas.2025.103775

**Published:** 2025-06-18

**Authors:** Jonathan X. Fang, Brian P. O’Neill, Tiberio M. Frisoli, James C. Lee, Gennaro Giustino, Pedro Engel Gonzalez, William W. O’Neill, Pedro A. Villablanca

**Affiliations:** a Center for Structural Heart Disease, Henry Ford Health System, Detroit, Michigan, USA; b National Heart Centre Singapore, Singapore; c Gagnon Cardiovascular Institute, Atlanta Health, New Jersey, USA

**Keywords:** aorta, aortic valve, peripheral vascular disease, valve replacement

**Figure:**
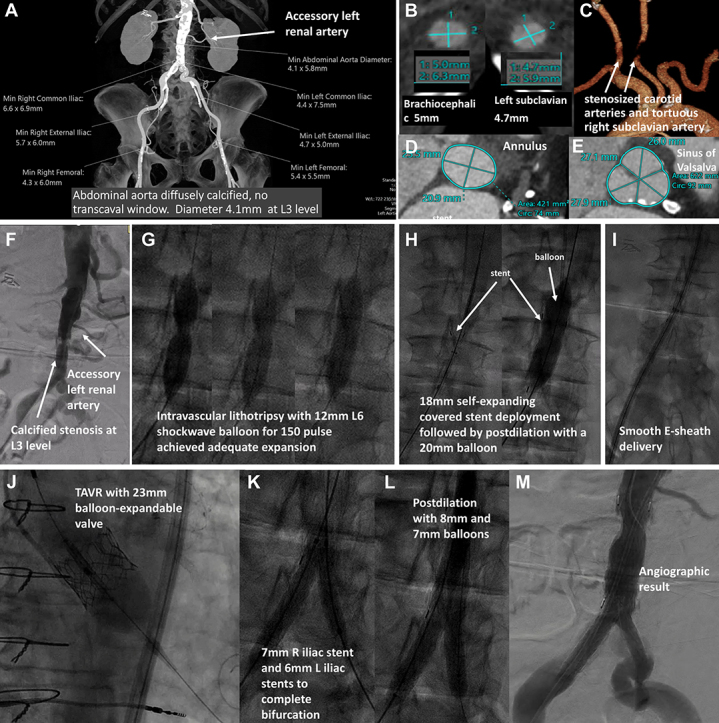

An 84-year-old female with severe aortic stenosis and NYHA functional class III symptoms with history of atrial fibrillation, coronary artery disease, diabetes, hypertension, hyperlipidemia and a Society of Cardiothoracic Surgeons Predicted Risk of Operative Mortality of 14.1% was evaluated for aortic valve replacement. She had a concomitant bilateral lower limb Rutherford class IIb claudication. Computed tomography (CT) revealed a severely calcified and stenotic aorta of diameter 4.5 mm at the level of the L3 vertebra and an accessory left renal artery. Bilateral femoral artery diameters were 6.0 mm and 5.5 mm on the right and left side, respectively. She had small and tortuous right subclavian and left brachiocephalic arteries of 5mm and 4.7 mm diameter respectively (Figures 1B and 1C) as well as bilateral carotid artery stenosis. The aortic annulus area and sinus of Valsalva dimensions were 421 mm^2^ and 26.0 × 27.1 × 27.9 mm respectively (Figures 1D and 1E). Given the lack of alternative access options and concurrent claudication, the heart team decided to perform transcatheter aortic valve replacement (TAVR) with concurrent treatment of abdominal aortic stenosis. Aortogram (Figure 1F) and intravascular ultrasound were obtained with a 14-F sheath via right femoral artery access, preclosed with 2 suture-based closure devices. Over a 0.035-inch wire, a 12-mm L6 lithotripsy balloon (Shockwave Medical) was passed over the stenotic aortic segment followed by lithotripsy with 150 pulses to achieve adequate expansion of the calcified stenosis (Figure 1G, Video 1), followed by placement of an 18-mm ovation covered stent (Endologyx) and postdilatation with a 20-mm balloon (Figure 1H) sized by intravascular ultrasound to facilitate the delivery of a 14-F E-sheath smoothy (Figure 1I). TAVR was performed with 23-mm balloon-expandable transcatheter heart valve at +1.5 mL volume to achieve a 5% oversizing based on CT measurements. (Figure 1J). Predilation was done with a 24-mm balloon based on CT measurements to facilitate transcatheter heart valve delivery owing to critical stenosis of aortic valve with an area of 0.47 cm^2^ and a peak/mean aortic valve gradient of 82/53 mm Hg measured on echocardiography. Given the presence of bifurcation disease, the abdominal stent was then completed by stenting the iliac bifurcation with 7-mm and 6-mm lifestream stents (Becton Dickinson) on the right and left, respectively, delivered via right femoral access and left radial access (Figure 1K), followed by kissing-balloon inflation with 7-mm and 6-mm balloons (Figure 1L) with good result (Figure 1M). The patient’s dyspnea and claudication subsided.

**Take-Home Message**

• With the use of a newer-generation lithotripsy balloon, lithotripsy-facilitated access of a severely calcified and stenotic abdominal aorta for transcatheter aortic valve replacement is feasible.

**Figure 1:**
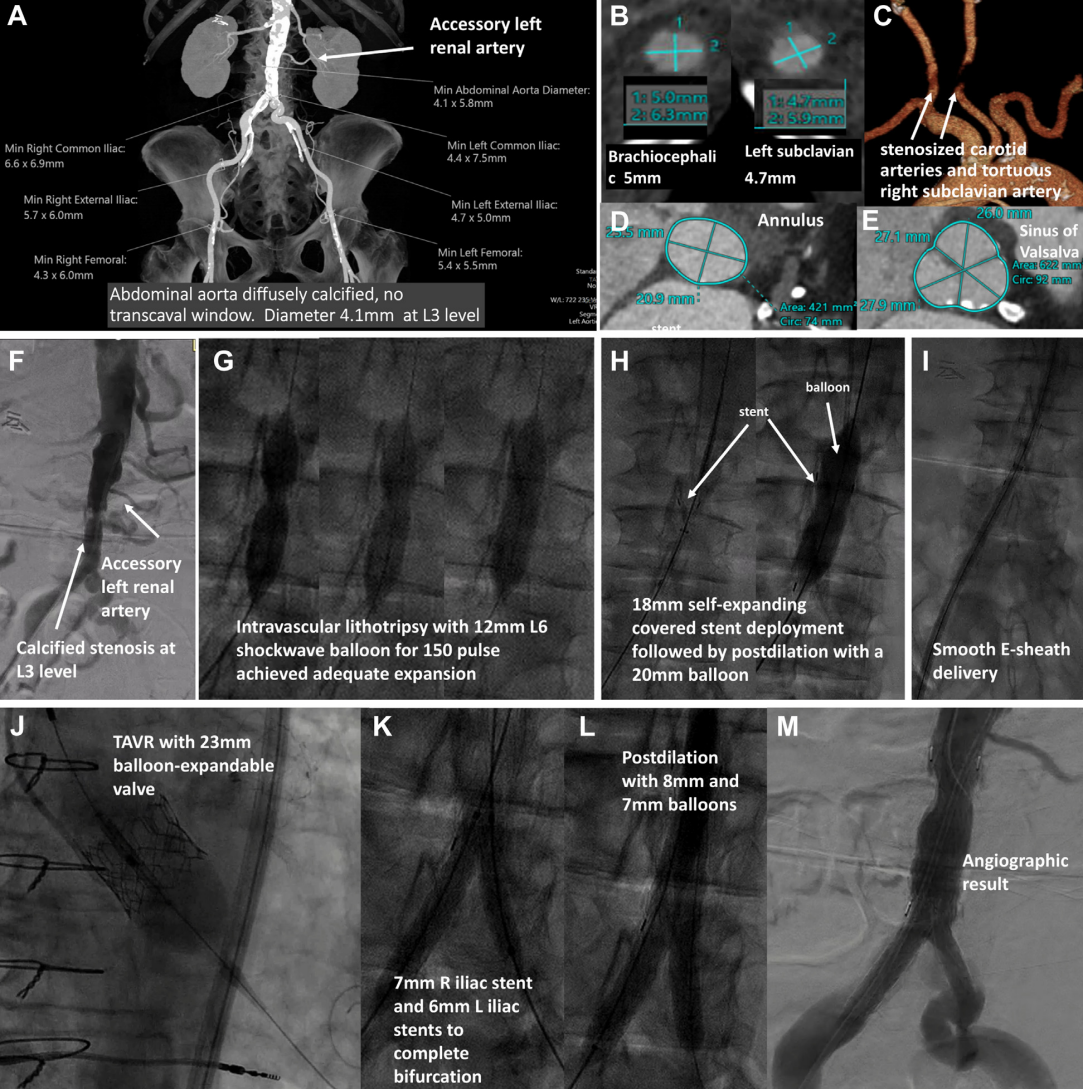
Workup and Procedure (A) Computed tomography of aorta and iliofemoral vessels, (B) brachiocephalic artery and right subclavian artery diameter, (C) bilateral carotid stenosis, (D and E) aortic annulus and sinus of Valsalva dimensions. (F) Angiogram showing abdominal aorta stenosis at L3 level and an accessory left renal artery. (G) Lithotripsy with a 12-mm L6 balloon for 150 pulses led to adequate expansion of the calcified stenosis. (H) Placement of a 18-mm covered stent followed by postdilation with a 20-mm balloon. (I) Smooth delivery of the E-sheath. (J) TAVR with 23-mm balloon expandable valve at +1.5 mL. (K and L) Completion of stenting iliac limbs and kissing balloon inflation. (M) Angiographic result. TAVR = transcatheter aortic valve replacement.

Lithotripsy-facilitated transfemoral access has been used for iliofemoral disease. The L6 lithotripsy balloon, available since 2023, delivers sonic energy evenly over a 30-mm length. With good vessel wall apposition, a 12-mm balloon can deliver the sonic energy deep into the vessel wall to treat deep calcifications in the aorta. To our knowledge, this is the first reported case of aortic lithotripsy-facilitated TAVR with concurrent stenting. We used cover stents prophylactically to lower the risk of perforation from passage and withdrawal of the E-sheath. An angiographic roadmap is crucial for avoiding inadvertent coverage of side-branches during deployment of the stents.

## Funding Support and Author Disclosures

Dr B.P. O’Neill is a consultant to and receives research support from Edwards Lifesciences. Dr Frisoli is a proctor for Edwards Lifesciences, Abbott, Boston Scientific, and Medtronic. Dr Lee is a consultant of Edwards Lifesciences. Dr W. O’Neill has served as a consultant for Abiomed, Edwards Lifesciences, Medtronic, Boston Scientific, Abbott Vascular, and St. Jude Medical and serves on the Board of Directors of Neovasc Inc. Dr Villablanca is a consultant for Edwards Lifesciences and Teleflex. Dr Giustino is a consultant for Edwards Lifesciences. All other authors have reported that they have no relationships relevant to the contents of this paper to disclose.

## Appendix

Video 1 Procedural Overview

